# A Qualitative Assessment of Mothers' Experience With Pediatric Anemia Care in Arequipa, Peru

**DOI:** 10.3389/fpubh.2020.598136

**Published:** 2020-12-16

**Authors:** Paola Louzado-Feliciano, Brianna Vargas, Madhavi Dandu, Shannon Fuller, Nicole Santos, Ángela Quiñones, Holly M. Martin, Alberto J. Caban-Martinez

**Affiliations:** ^1^UCSF Institute for Global Health Sciences, San Francisco, CA, United States; ^2^Department of Public Health Sciences, University of Miami Leonard M. Miller School of Medicine, Miami, FL, United States; ^3^UCSF School of Medicine, San Francisco, CA, United States; ^4^Catholic University of Santa Maria, Professional School of Human Medicine, Arequipa, Peru; ^5^Sylvester Comprehensive Cancer Center, University of Miami Leonard M. Miller School of Medicine, Miami, FL, United States

**Keywords:** pediatrics, anemia, malnutrition, primary health care, South America

## Abstract

**Background:** Despite national efforts to control pediatric anemia in Peru, each year, 67.4% of all newborns are diagnosed with anemia during their first year of life. The literature on Peruvian mothers' understanding and beliefs of pediatric anemia is limited. In the present study, we aimed to understand mother's perspective of pediatric anemia and explore their perceptions on how to prevent and treat anemia in Peru.

**Methods:** During May–June 2018, we administered a short demographic questionnaire and conducted language-sensitive interviews with mothers of children clinically diagnosed with anemia in three different governmental health centers in Arequipa, Peru. Interviews were audio-recorded and transcribed verbatim. We used the Framework Analysis approach to analyze qualitative data.

**Results:** A total of 14 Peruvian mothers were interviewed. Across interviews, three main themes emerged: (I) Mothers' Understanding of Pediatric Anemia; (II) Attitudes about Provider Recommendations for Pediatric Anemia Control; and (III) Barriers to Effective Pediatric Anemia Control. Peruvian mothers expressed skepticism toward national pediatric anemia guidelines as they believe recommendations received at health clinics jeopardized their children's overall health. Participants identified several barriers to effective anemia control: limited and confusing health information received during pediatric healthcare appointments, lack of systematic protocols in health clinics, and inconsistent referral processes.

**Conclusion:** We identified factors that limit the acceptance of current pediatric anemia control guidelines followed at governmental health centers in Arequipa, Peru. Understanding maternal beliefs concerning pediatric anemia can guide future anemia control guidelines at the primary care level for pediatric patients in Peru.

## Introduction

Anemia is a medical condition associated with an insufficient number of red blood cells or their capacity to carry oxygen in blood (1, 2). Anemia affects millions of children worldwide and can weaken a child's immune system leading to adverse health effects (3). Currently, the leading cause of anemia is iron deficiency, accounting for half of all anemia reported cases (1, 4). Suffering from iron deficiency anemia during childhood can lead to cognitive and motor deficiencies which can lead to difficulties with academic achievement and later health and well-being including productivity (2, 5, 6).

In Peru, it is estimated that 404,938 out of 600,000 yearly live births will be diagnosed with iron deficiency anemia during their first year of life (6, 7). Due to this high burden, controlling pediatric anemia is a top priority for Peru's Ministry of Health (MINSA) and Ministry of Development and Social Inclusion (MIDIS) (8–11). MINSA and MIDIS have been collaborating with regional government's health departments, community health workers, and health care practitioners to prevent and control pediatric anemia (10, 11). All health establishments (public or private) in Peru must follow health guidelines established by MINSA, such as the *Prioritized Interventions according to Life Cycle for Anemia Control* (12, 13). These interventions focus on iron supplementation; iron-folic acid (IFA) supplementation during pregnancy and ongoing IFA supplementation 30 days post-partum; iron drops (ferrous sulfate) for infants 0–6 months; and, micronutrient supplements for infants 6–36 months (8). Nonetheless, despite national anemia prevention efforts, data published by Peru's National Institute of Computing and Statistics suggest pediatric anemia prevalence rates are stagnant (14).

Peru's National Institute of Health suggested research is needed to better understand what other factors, despite iron deficiency might be contributing to the ongoing pediatric anemia burden (15). Factors such as parental understandings, cultural traditions, and social representations might affect health care preventive or treatment intervention adherence (16). Thus, impacting the effectiveness of anemia nutrition-based interventions at a national level. These factors might not be taken into consideration when developing interventions to tackle pediatric anemia in Peru. As a formative first step to address the burden of pediatric anemia in Peru, we aimed to understand Peruvian mother's perspective of pediatric anemia and explore their perceptions on how to prevent and treat pediatric anemia in Peru.

## Methods

### Study Design

We used qualitative research methods to explore Peruvian mother's perceptions of pediatric anemia in Peru. We gathered data from a demographic questionnaire and an in-depth interview in order to assess our research aims. We identified themes regarding beliefs, attitudes, facilitators, and barriers among mothers of children diagnosed with anemia. The study research protocol was reviewed and approved by University Institutional Review Board (IRB) and local research review board in Arequipa, Peru.

### Study Setting

This study was conducted across three governmental health centers from two different districts located in the peri urban regions of Arequipa, Peru. We partnered with governmental health centers as they are the primary providers of health in Peru (17, 18). Patients seen at public health centers have access to free healthcare and available medications at the health centers (17). All health centers (public or private) in Peru must follow protocols established by the Peru's Ministry of Health such as the *Prioritized Interventions according to Life Cycle for Anemia Control* (8, 12, 13). Anemia prevention efforts for children focus on iron supplementation; IFA supplementation when a woman is pregnant and ongoing IFA supplementation 30 days post-partum; iron drops for infants 0–6 months; and, micronutrient supplements for infants 6–36 months (8, 12, 13). Micronutrient supplements are meant to be given for a period of 12 months after the child reaches 6 months (8, 12, 13). As for pediatric anemia treatment, the standard is to give infants iron drops/syrup for a period of 6 months after a diagnosis is delivered and to use the micronutrient supplement powder packets provided by health care establishments (8, 12, 13). The Ministry of Health also recommends parents provide a diet that incorporates iron rich foods, preferably from animal origin such as animal blood, spleen, heart, and liver after the child reaches 6 months of age (8, 12, 13).

### Participant Recruitment

During May–June 2018, we used a purposeful sampling approach to recruit participants for our study. Our team coordinated in-person health center visits, explained the study's purpose to eligible participants during medical appointments or house visits with the health center's nutritionist and subsequently invited mothers for an interview. Eligibility criteria included: ≥18 years of age; mothers of a child aged ≤5 years of age that had received a clinical anemia diagnosis within the last year of life and speaks or writes in English or Spanish. Our study focuses on maternal perspective of pediatric anemia as mothers are the primary child caregivers in Peru. Participants did not receive an incentive to participate in our interviews.

### Participant Consent and Data Collection Procedures

We collected data *via* a one-time, language-concordant, paper-based demographic questionnaire, and an in-depth interview which on average lasted 30 min. Both data collection instruments (i.e., demographic questionnaire and interview guide) were reviewed and approved by our regional government partners before administration. Prior to engaging in any research activity, all participants consented in their native language. The language-concordant questionnaire consisted of a mix of 10 open and closed-ended questions. The questionnaire gathered demographic characteristic from the mothers (i.e., participant age, sex, birthplace, education level, and the total number of biological children) and characteristics about their anemia diagnosed child (i.e., current child age, date of anemia diagnosis, hemoglobin level at diagnosis, and current anemia status). Mothers completed the paper-based questionnaire independently, researchers were present in case participants needed clarification on a question. Upon completion of the demographic questionnaire, the interview began. Interview domains focused on five main inquiry concepts: (1) general understanding of anemia, (2) pediatric anemia prevention beliefs, (3) pediatric anemia treatment and use, (4) barriers to anemia prevention and treatment, and (5) facilitators to anemia prevention and treatment. All interviews were conducted in Spanish, audio-recorded and saved on electronic devices. Two Spanish researchers led this process; one conducted the interviews in Spanish while the other researcher took field notes with the purpose of complementing data collection and analysis. After each interview ended, the researchers reflected on field notes and expanded on points not reflected on the voice recording such as participant non-verbal behavior. Researchers did not use a template to record field notes. Data collection ceased once data saturation was reached.

### Data Analysis for Quantitative and Qualitative Data

Quantitative data was analyzed with SPSS (Version 26.0) to provide descriptive statistics. Data collected from the interview transcripts were first transcribed in Spanish and then translated to English by a bilingual researcher. The researcher compared English and Spanish transcripts to ensure meaning was not lost in the English transcript. After translation and transcription processes were completed, data was analyzed with a framework method approach (18–20). We used an emergent code system as this allowed codes to emerge from the data collected organically. The first set of interviews were coded independently with the purpose of applying potential labels or codes to the second set of interview transcripts. After allowing codes to emerge organically, a final thematic framework was established; each code was given a unique definition and final codes were grouped by categories. Indexing was accomplished by applying the final thematic framework to the transcriptions using an online software called Dedoose (Version 8.0.35). After indexing was completed, we charted the data by developing a framework analysis table using a matrix. Once data was charted, mapping and interpretation began. This last phase involved an analysis of the key characteristics and emerged themes from the framework table. The framework method allowed us to describe participants' perceptions of the issue being studied in Peru. We sought patterns such as: similarities, differences, sequences, correspondence to other activities, or events and causation (21).

## Results

### Participant Demographics

A total of 14 Peruvian mothers were interviewed. The participant sample had a group mean age of 30 ± 7.1 years, of which 50% had lived in Arequipa for more than 20 years, 50% had attended middle school, and 64.3% had two biological children or more (Table 1). At the time of interview, children with a clinical anemia diagnosis of mothers participating in our study had an average age of 11.1 ± 6.7 months, of which 64.2% still had anemia, 64.2% were patients at the “Villa de Jesus” health center, and 28.5% were diagnosed with anemia at ≤4 months of age (Table 1).

**Table 1:** Characteristics of Peruvian mother's interviewed and their child with a clinical anemia diagnosis (*n* = 14), Arequipa, Peru, May–June 2018.

**Mother's characteristics**	**Total sample *n* = 14^**†**^**
**Age**	
18–29 years old	2 (14.3)
30–39 years old	8 (57.1)
40–49 years old	4 (28.3)
**Gender**	
Female	14 (100.0)
Male	0 (0)
**Educational attainment**	
Less than high school	10 (71.4)
High school	2 (14.3)
Greater than high school	2 (14.3)
**Number of mother's biological children**	
One	5 (35.7)
Two	4 (28.6)
Three or more	5 (35.7)
**Characteristics of children clinically diagnosed with anemia**	
**Government health centers**	
Villa de Jesus	9 (64.2)
Zamacola	3 (21.4)
Ciudad Blanca	2 (14.2)
**Child's hemoglobin (grams/dl) level at diagnosis**	
Mild anemia (10.0–10.9)	13 (92.9)
Moderate anemia (7.0–9.9)	1 (7.1)
Severe anemia (<7.0)	0 (0)
**Child's age at anemia diagnosis (months)**	
≤4 months	4 (28.5)
5–6 months	8 (57.1)
>6 months	2 (14.3)
**Child's anemia status (at time of the interview)**	
Currently has anemia	9 (64.2)
No longer anemic	3 (21.4)
Unknown	2 (14.2)

*Differences in sub-total population sample due to item non-response or missing*.

### Qualitative Findings

Seven subthemes emerged from our analysis which were organized into three main overarching themes: (I) Mothers' Understanding of Pediatric Anemia; (II) Attitudes about Provider Recommendations for Pediatric Anemia Control; and (III) Barriers to Effective Pediatric Anemia Control. Table 2 provides a brief summary of the main themes and subthemes to be discussed. Participants identifiers = (Interview ID, Mothers age at the time of interview).

**Table 2:** Summary of key themes and subthemes, key informant interviews (*n* = 14), Arequipa, Peru May–June 2018.

**Themes**	**Subthemes**
I. Mothers' understanding of pediatric anemia	1st subtheme: Association of pediatric anemia as a malnourishment consequence
II. Attitudes about provider recommendations for pediatric anemia control	2nd subtheme: Skepticism toward micronutrient supplements
	3^rd^ subtheme: Concerns about ferrous sulfate
	4^th^ subtheme: Preference to prevent and treat pediatric anemia with a well-balanced diet
III. Barriers to effective pediatric anemia control	5^th^ subtheme: Limited Health Information from Health Providers
	6^th^ subtheme: Inconsistent referral processes
	7^th^ subtheme: Lack of systematic treatment recommendations

#### Theme I: Mothers' Understanding of Pediatric Anemia

##### 1st Subtheme: Association of Pediatric Anemia as a Malnourishment Consequence

To conceptualize mothers' understanding of pediatric anemia, we initially asked participants what pediatric anemia meant to them and what came to mind when we mentioned the word anemia. Across all interviews, mothers associated pediatric anemia as a malnourishment consequence, it was common for mothers to state, “*anemia means lack of iron, vitamins, or having a poor diet*.” One participant said, “*Anemia means that my baby is poorly fed, the baby is malnourished, and due to that, the baby has anemia” (1, 31)*. Another participant mentioned, “*Anemia is lack of proper nutrition; I suppose not enough iron” (12, 38)*. This perception was associated with how health providers—nurses, medical doctors, or nutritionists—in the government health centers explained the cause behind their child's anemia was due to a lack of following a well-balanced diet that also incorporated iron-rich foods, “*The lady [referring to the health center nurse] told me anemia was caused because we do not give babies the proper alimentation; for not giving babies things such as animal blood which increases iron […]” (14, 37)*. Participants expressed how this association of anemia with malnourishment was a common thing to hear during the medical appointments, “*He [referring to the pediatrician] told me that I was not feeding him [participant's baby] a well-balanced diet and that this was the reason why my baby had developed anemia. He [referring to the pediatrician] told me to give him liver and blood” (4, 25)*.

Mothers mentioned health providers explained another possible reason behind the diagnosis was the mothers' diet, as breastmilk was the child's primary source of food, “*In the case of my baby, he was diagnosed at six months, he still did not eat food, he was breastfeeding exclusively. If I ate bad food, I did not have enough nutrients […]” (10, 22)*. This information often caused mothers to blame themselves for their child's anemia diagnosis. They believed they were not providing enough nutrients for their children*, “Sometimes I believe, my baby got sick because of me, maybe I did not eat well” (2, 20)*.

#### Theme II: Attitudes About Provider Recommendations for Pediatric Anemia Control

##### 2nd Subtheme: Skepticism Toward Micronutrient Supplements

Mothers only talked about micronutrient supplements as a preventive measure when asked directly by the interviewer, “*what do you believe are micronutrient supplements.”* Most participants explained micronutrient supplements are recommended by the government health centers when their baby reaches 6 months. Even though participants were aware of micronutrient supplementation as a way to prevent pediatric anemia, this was not their preferred method to prevent pediatric anemia.

Interviewer: *Could you mention examples of preventive methods so that anemia does not develop in a baby?*
Participant (5, 29): *Following a well-balanced diet, for me, that is enough*.
Interviewer: *Could you explain what micronutrient supplements are?*
Participant (5, 29): *Micronutrients have zinc and iron; it is like a supplement that is added to a baby's food*.

Mothers expressed doubt and skepticism regarding the health benefits of the government health centers' micronutrient supplements. Interviews showed participants did not have an overall positive attitude toward using daily micronutrient supplementation. Mothers stated that micronutrient supplements suppressed their baby's appetite and caused constipation: “*I was giving micronutrient supplements to my babies, but I stopped 15 days ago because it was giving my babies constipation's, which is why I stopped giving the supplements to my babies” (1, 31)*. Another mother expressed, “*I have only given a little [referring to micronutrients] to my baby because when my [older] daughter was taking them; she lost her appetite” (3, 25)*.

Participants also appeared to be influenced by relatives or friends who informed them micronutrient supplements should not be given to babies as they have side effects that would make their babies sick: “*I have not given my baby micronutrient supplements, because people have told me, it will give my baby diarrhea and that the supplements make them [referring to babies] get sick. I did not want my baby to lose weight” (9, 22)*. This collective idea of micronutrients causing negative side effects (i.e., diarrhea, loss of appetite, or constipation) influenced the majority of participants to stop following or not follow at all the micronutrient supplementation recommendations given by their providers.

##### 3rd Subtheme: Concerns About Ferrous Sulfate (Iron Drops)

Participants indicated ferrous sulfate is the primary treatment prescribed when a child is diagnosed with anemia at a government health center. However, mothers expressed concerns about utilizing ferrous sulfate. Mothers brought up the question of alleged health benefits of ferrous sulfate vs. the side effects it could cause. This medication was described as “*not being very effective for treating anemia due to its side effects.”* One mother mentioned, “*It was not very efficient. I think it caused more harm than good because it caused my baby constipation even though I was giving my baby a lot of water” (10, 22)*. Additionally, mothers who had previously used ferrous sulfate medication with their older children that experienced adverse side effects mentioned that they decided to forego the medication with their current anemia diagnosed child. When asked about her opinion concerning ferrous sulfate, a participant shared, “*I was not going to give him the ferrous sulfate because my baby was going to get worse, it was going to take away his appetite, and the ferrous sulfate was not going to raise his hemoglobin. Instead, I gave him ferranin forte” (6, 29)*. Participant (6, 29) was not the only mother who mentioned using an alternative anemia treatment medication not recommended by the government health center providers.

Participants talked about two alternative anemia medications, (1) “*ferranin forte”* and (2) “*emociton.”* These medications were either recommended to mothers by personnel in pharmacies or providers at a private health center. The medicines were used in the same way as ferrous sulfate as it came in drop or syrup form. Mothers indicated “*ferranin forte”* or “*emociton”* medication worked better than ferrous sulfate as it appeared to be more effective in treating anemia, “*Emociton is stronger than ferrous sulfate and does not cause constipation. This medication does not affect my baby's stomach” (10, 22)*.

##### 4th Subtheme: Preference to Prevent and Treat Pediatric Anemia With a Well-Balanced Diet

We found mothers' idea of anemia prevention and treatment overlapped as they believed pediatric anemia could be prevented and treated by following a well-balanced diet that incorporated iron-rich foods. When asked how pediatric anemia can be prevented, one mother said*, “Anemia can be prevented by giving the baby foods such as liver, spleen and lentils.liver is what we should give babies the most” (6, 29)*. When we asked the same mother how pediatric anemia can be treated, she said, “*Anemia can be treated with nutrition, especially lentils, liver, and animal blood” (6, 29)*.

Mothers stated that an iron-rich diet was their preferred method to treat and prevent anemia. It was easy to follow as it consisted of preparing meals for their babies and not having to buy medication. Mothers also explained that following a well-balanced diet with a focus on iron-rich foods was recommended by their friends and family members. One mother said: “*My family told me that to prevent anemia, you have to consume a lot of liver, which has iron” (7, 40)*. This perception was common across interviews; participants usually mentioned their “*mothers”* recommended foods such as “*spleen, liver, heart, lentils, and animal blood”* to prevent or treat anemia.

#### Theme III: Barriers to Effective Pediatric Anemia Control

##### 5th Subtheme: Unclear and Limited Health Information Received From Health Providers

Mothers expressed receiving confusing health information from their health care providers, which impacted their child's treatment. In some cases, mothers were told their children did not have a severe enough vs. a severe enough anemia and health providers did not explain well how to treat it because “*the baby was not that sick.”* Participant (10, 22) recommended that nurses be more careful when explaining lab analyses or certain medications, “*Nurses should be more careful, sometimes they are careless. They [nurses] told me my baby did not have very low hemoglobin, so I did not treat my baby as I should have when he was first diagnosed with anemia”* (10, 22) and to increase the availability of health providers in the health centers, “*health centers should have nutritionists available so that they can explain to us more” (13, 35)*.

Mothers also shared they wished health centers provided more health education “…* we should have health talks, more health education….”* It was also typical for participants to share how they wish they understood more about pediatric anemia, “*I understand anemia is low iron in the blood, the problem is that I don't know how extreme anemia consequences can be” (13, 35)*, or the reasoning behind their child's diagnosis, “*I want to know how anemia develops, I don't understand when my baby was born, she was okay. Now when she reached six months and is supposed to start eating, I found out she had anemia” (9, 22)*.

##### 6th Subtheme: Inconsistent Referral Processes

Participants' descriptions of operational practices within the health centers showed inconsistency in the general guidelines followed by health care providers. Instructions regarding anemia diagnosis protocols appeared to be unorganized and arbitrary. Participants explained that during their child's monthly medical appointments at the health center, parents are supposed to be referred by the health center's nurse to either the on-call general doctor, a pediatrician (if the health center has one) or the nutritionist. Referral from nurse to the doctor depends on what a lab analysis shows or the symptoms the babies are presenting with. Nonetheless, participants shared how sometimes they were not referred to the proper medical provider (i.e., health center's general doctor, pediatrician, or nutritionist), which impacted the treatment their babies needed at the moment. Participant (10, 22), shared,

“*At six months, nurses did not pass me to general medicine to receive anemia consult with the general doctor; at 8 months, it was another nurse that did. The first nurse told me that with micronutrient supplementation, the baby was going to get better as the anemia was not severe. Later, at eight months, another nurse told me the first nurse should have passed me to general medicine to get a prescription of ferrous sulfate. The second nurse told me, "you have to go to general medicine because your baby does have anemia.”*

##### 7th Subtheme: Lack of Systematic Treatment Recommendations

Moreover, guidelines for anemia treatment also appeared to be arbitrary. Some participants stated health providers recommended the use of micronutrient supplements as anemia treatment, “*I was told to give my baby ferrous sulfate plus the micronutrient supplements” (9, 22)*, while other participants stated they were advised not to give the child micronutrient supplements while the child was recuperating from anemia, “*I will start giving my baby micronutrient supplements again when my baby's hemoglobin goes back to normal because when the baby has low hemoglobin, you cannot give the baby micronutrient supplements” (6, 29)*.

## Discussion

In order to understand potential factors affecting the pediatric anemia burden in Peru, we sought to understand Peruvian mother's perspective of pediatric anemia and explore their perceptions on how to prevent and treat pediatric anemia in Peru. From the 14 mothers interviewed, 13 mothers knew about their child's anemia diagnosis for at least 1 month. However, their understanding of anemia revolved around cause. Mothers mainly believed anemia is just a consequence of malnourishment and failed to define anemia as a medical condition. Consequently, mothers preferred method for anemia prevention and treatment was following a well-balanced diet with a high consumption of iron-rich foods. This lack of understanding can be one of the reasons as to why mothers demonstrated negative attitudes toward utilizing a prevention measure such as micronutrient supplementation and a treatment measure such as ferrous sulfate. Mothers seemed to have preconceived notions of the use for both of these interventions as their perception revolved around how these interventions caused more harm than good. Mayca-Pérez et. al. (22) also showed that parents from Awajún and Wampis in Peru focused on diet to control their children's anemia instead of utilizing the available iron and micronutrient supplements provided from health centers in their communities. This situation exemplifies a gap between parents and the health information received in government health centers, and how communication between the caregiver and provider is an essential component for adherence to preventive and treatment interventions for the pediatric population. Published studies have shown the importance of patient-provider communication as poor patient-provider communication has been proven to lead to poor treatment adherence (23, 24). In this case, poor caregiver-provider communication could compromise the treatment a child with anemia needs to receive if the caregiver does not fully comprehend the health consequences of the medical condition. Furthermore, 50% of the mothers interviewed had only middle school education, indicating a low socio-economic background. Research has shown how poverty is a key determinant of adherence to prevention and treatment interventions (25, 26). Poverty can be an underpinning reason as to why mothers had a limited understanding of anemia, which could impact their attitudes toward the current pediatric anemia control guidelines followed at governmental health centers in Arequipa, Peru.

Peru's national guidelines for anemia control involves iron drops for infants 0–6 months, micronutrient powder supplements for infants 6–36 months, and ferrous sulfate for infants/older children diagnosed with anemia (8). These guidelines are meant to be followed in all health establishments of Peru. However, mothers did not indicate following the guidelines mentioned. This suggests pediatric patients from government health centers might not have proper access to iron supplementation during 0–36 months. Findings presented by Amerson et al. (27) indicated that only 12% of children in Peru might benefit from iron supplementation government programs due to system limitations. If mothers do not follow these guidelines, it has the potential to increase a child's chance to be diagnosed with anemia, as the child did not receive iron supplementation since birth (3, 28).

We also identified arbitrary use of anemia control standards. Some providers instructed parents to stop the use of micronutrient powder supplements until the baby's hemoglobin level reached an acceptable hemoglobin threshold. In other cases, providers informed parents to complement their child's anemia treatment of ferrous sulfate with ongoing micronutrient supplementation. This arbitrary use of guidelines gives space for inadequate anemia treatment as micronutrient powder supplements and ferrous sulfate do not contain the same ingredients (12). Furthermore, the misuse of anemia control standards might exacerbate the prevalence of pediatric anemia in Arequipa as anemia control is compromised. If parents are not taking the necessary precautions to control anemia during their children's first years of life this may lead to an ongoing micronutrient deficiency which can weaken their immune system and have a lasting effect on a child's growth and development (3).

### Limitations

While these findings are critical for the pediatric population in Peru, it is not without limitations. Our research study was developed with the goal to explore and understand the perspectives and experiences of a unique population with a specific background in a particular setting; thus, this limits its transferability. It is also worth noting, our study did not receive the paternal perspective as only mothers participated in the research. Additionally, the study sample included 14 participants from government health centers in the peri urban area of Arequipa; it is possible that our sample might not be representative of mothers' perspective throughout private health centers or centers located at an urban setting in Arequipa. Despite limitations, this study has several strengths. This is the first research study to look at maternal perceptions of pediatric anemia in government health centers of Peru. It focuses on a current public health crisis as more than half of all children born in Peru will be diagnosed with anemia during their first year of life. Furthermore, this research provides evidence on barriers identified by the caregivers of the pediatric patients in government health centers, which can be taken into account when creating or modifying anemia control standards.

## Conclusions and Implications

This research illustrates how mothers of anemic children conceptualize and manage anemia which plays a vital role in their adherence to treatments recommended by health providers in Arequipa, Peru. This study provides evidence on maternal perceived barriers to the use of current anemia prevention and treatment measures available for their children which can guide health care providers into filling the gaps participants identified for proper pediatric anemia treatment and prevention. Disseminating these findings to MINSA, Peru's Public Health Department, government health centers providers, and policymakers is of high importance. Our evidence could have a positive impact on the development of future pediatric anemia healthcare guidelines in Peru. Future research should focus on collecting data from different jurisdictions throughout Peru including both public and private health centers so that we can better conceptualize clinical practice and policy guidelines utilized during pediatric anemia cases.

## Data Availability Statement

The quantitative raw data supporting the conclusions of this article can be made available by the authors, without undue reservation.

## Ethics Statement

The studies involving human participants were reviewed and approved by University of California San Francisco (UCSF) Institutional Review Board (#18-24544) and the Peru Red de Salud Arequipa-Caylloma Executive Committee. The ethics committee waived the requirement of written informed consent for participation.

## Author Contributions

PL-F, ÁQ, and HM conceived the study, participated in its design, and coordination. PL-F, BV, ÁQ, and HM collected field data, entered study data, assisted in data analysis, and interpretation of study results. PL-F, BV, MD, SF, NS, AC-M and HM performed final analyses and co-drafted the manuscript. All authors read, revised, and approved the final manuscript.

## Conflict of Interest

The authors declare that the research was conducted in the absence of any commercial or financial relationships that could be construed as a potential conflict of interest.
